# Preliminaries to a Psychological Model of Musical Groove

**DOI:** 10.3389/fpsyg.2019.01228

**Published:** 2019-06-04

**Authors:** Olivier Senn, Dawn Rose, Toni Bechtold, Lorenz Kilchenmann, Florian Hoesl, Rafael Jerjen, Antonio Baldassarre, Elena Alessandri

**Affiliations:** School of Music, Lucerne University of Applied Sciences and Arts, Lucerne, Switzerland

**Keywords:** music, groove, entrainment, dancing, pleasure, body movement, sensorimotor synchronization

## Introduction

Humans often feel motivated to move their bodies in response to music; this experience is generally referred to as “feeling the groove.” In this paper we discuss ideas about how the experience of groove can be modeled from a psychological point of view.

As a musical term, groove was originally coined in the context of Western popular music (Pfleiderer, 2006, p. 297ff; Abel, 2014, p. 18) where it has several meanings. It may refer to a repeated pattern that represents the basis of a piece (“a groove,” Zbikowski, 2004). It may also denote the temporal interaction and effortless synchronized performance within a band (“to groove,” Berliner, 1994, p. 388; Keil, 1995; Doffman, 2008, p. 11; Hosken, 2018), or the power of music to provoke body movement in listeners (Roholt, 2014, p. 85).

Music psychology builds on the last of these meanings and defines groove as a person's urge to move in response to music, accompanied by a feeling of pleasure (Madison, 2001, 2006; Janata et al., 2012). The psychological concept of groove is not restricted to Western popular music. It can be applied to any situation in which music triggers body movement, regardless of style or cultural background (Pressing, 2002). Since music is used for dancing in a majority of cultures (Kaeppler, 2000; Nettl, 2000), it is not surprising that there are concepts similar to groove in several languages, for example “balanço” in Brazilian (Vurkaç, 2012), “nori” in Japanese (Kawase and Eguchi, 2010), or “lüpfig” in Swiss German (Ringli, 2006, p. 123).

Empirical studies of groove have focused on musical properties that may have an effect on the groove experience. Some studies have investigated qualities that add interest to the music, such as syncopation (Sioros et al., 2014; Witek et al., 2014), rhythmic variability (Wesolowski and Hofmann, 2016), microtiming (Davies et al., 2013; Frühauf et al., 2013; Senn et al., 2016; Hofmann et al., 2017), or the interaction of rhythmic and harmonic complexity (Matthews et al., 2019). Other studies focused on properties that emphasize the regularity of the meter, such as beat salience (Madison et al., 2011) or tempo (Etani et al., 2018). Listeners' personal background (e.g., musical taste or familiarity with the repertoire) has also been found to influence the groove experience (Janata et al., 2012; Senn et al., 2018, 2019).

Since musical features, styles, surveyed populations, methods, and results differ greatly across empirical groove studies, it is a challenge to obtain a bigger picture of the progress made in the field. Therefore, this paper presents a psychological model of groove as a broad theoretical framework. This will enable the application of findings from groove studies to be considered from a wider perspective. Our model adopts Merker's (2014) idea that a person who experiences groove needs to have an inner representation of the music's temporal regularities, which allows for motor planning and synchronized body movement. The model also integrates Senn et al.'s (2018, p. 4) suggestion that music, in order to groove, must provide the listener with a motivation to move.

## Description of the Proposed Psychological Groove Model

**Figure 1:**
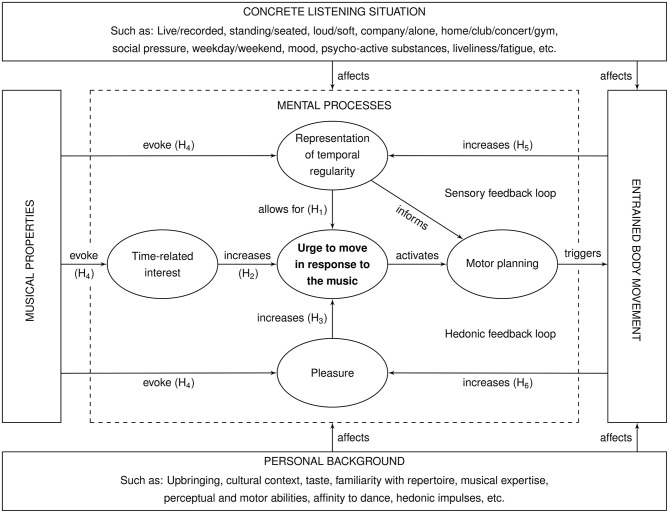
Psychological groove model with hypothesized effects (H_1_—H_6_).

Figure 1 presents a diagram of the hypothetical groove model. The central dashed box addresses the *mental processes* that are considered to be relevant to groove. These processes are triggered by the *properties of the music* (left), and they may lead to *entrained body movement* in the listener (right). The mental processes are influenced by the *concrete listening situation* (top) and the *personal background* of the listener (bottom).

### How Music Makes Us Want to Move

*Musical properties* (Figure 1, left) is an umbrella term for all possible ways of describing music. The descriptors may be purely acoustic (e.g., loudness, frequency spectrum). They may refer to specific sound patterns resulting from the process of music making (e.g., clave rhythm, guitar riff, tabla taal, bass line) or to more abstract concepts that describe the development of music in time (e.g., meter, rhythm, syncopation, riff, harmony, form). The music may come from any style or cultural background. Since musical properties change considerably across contexts, the model is agnostic with respect to pre-defining a selection of musical properties that are relevant to groove.

As music is perceived by a listener, it triggers three mental processes (Figure 1, dashed box) that we hypothesize to be instrumental in causing an *urge to move*:

— Listeners derive an inner *representation of temporal regularity* from the music (Large and Jones, 1999; Vuust et al., 2018). The regularity may be based on an isochronous pulse or tactus as in many Western popular music styles (Merker et al., 2009). Yet, an isochronous pulse is by no means the only way to create a stable (and thus predictable) temporal structure, as Polak et al. (2016) have shown with respect to Malian jembe drumming. Empirical groove studies have confirmed that temporally regular musical properties predict groove, such as the salience of the beat (Madison et al., 2011) or the tempo (Etani et al., 2018). The neural representation of temporal regularity has received considerable attention in neuroscience (for an overview, see Ivry and Spencer, 2004; Paton and Buonomano, 2018).
— The temporal organization of the music may raise listeners' *interest*, which represents one dimension of the aesthetic response to art (Cupchik and Gebotys, 1990). Time-related interest may for example be rooted in rhythmic complexity: syncopation (Sioros et al., 2014; Witek et al., 2014), event density (Madison et al., 2011), or the interaction of rhythmic and harmonic complexity (Matthews et al., 2019) make the time organization of the music more interesting, and they have been found to affect the groove experience.
— Listening to music causes people to experience *pleasure*, which is another core dimension of the aesthetic response (Cupchik and Gebotys, 1990). Listening to music can be a hedonic activity that is considered to be rewarding and pleasurable by itself (Berlyne, 1974, p. 8; Zatorre and Salimpoor, 2013). The role of *pleasure* in groove is not clear-cut: pleasure has been used to define groove in some cases (Madison, 2001; Janata et al., 2012; Witek et al., 2014; Senn et al., 2018). Yet, recently, Matthews et al. (2019) treated *pleasure* as a mediator for groove.

It is the primary goal of groove research to investigate the *urge to move in response to the music* and the circumstances under which it arises. We hypothesize that listeners' *representation of temporal regularity*, their *rhythm-related interest* and the *pleasure* they experience while listening are causally linked to the urge to move. Specifically, our hypotheses are:

— H_1_: The inner representation of temporal regularities in the music is a precondition for listeners' urge to move in response to the music.
— H_2_: Interest in the temporal organization of the music increases listeners' urge to move.
— H_3_: Listeners' pleasure while listening to the music also increases their urge to move.
— H_4_: Musical properties do not affect the urge to move directly, but they are mediated through the representation of temporal regularity, rhythm-related interest and listening pleasure.

### How Body Movement Affects Our Perception of Music

As a response to the *urge to move*, listeners' *motor planning* abilities may be activated (Zatorre et al., 2007; Wong et al., 2015) and map *entrained body movement behavior* onto the music. This process can be either conscious or subconscious (Phillips-Silver and Keller, 2012), and it can express itself as dancing, finger tapping or any other form of entrained movement (Clayton, 2012; Repp and Su, 2013; Burger et al., 2014; Ross et al., 2016).

We assume that *entrained body movement* activates two feedback loops:

— *Sensory feedback loop*: Expressing rhythmic processes through body movement can be understood as an instance of embodied perception (Wilson, 2002; Sebanz and Knoblich, 2010). As the person moves along with the music, the temporal regularities of the music are represented in various sensory systems in addition to hearing, such as touch (tapping, stomping, dancing), the vestibular system, and vision (head bobbing, dancing). Multimodal perception supposedly enhances the inner *representation of temporal regularities* (Spence and Driver, 2004). This in turn strengthens the *urge to move* and feeds back through *motor planning* to *entrained body movement*.
— *Hedonic feedback loop*: *Entrained body movement* is known to be a pleasurable activity by itself (Shaulov and Lufi, 2009). Moving in response to music has been found to increase the *pleasure* experienced by the listener (Bernardi et al., 2017). This closes a second feedback loop through *pleasure*, the *urge to move*, and *motor planning* back to *movement*. The hedonic feedback loop assumedly increases the individuals' endurance when repetitive movement tasks are synchronized with music, for example in sports (Karageorghis and Priest, 2012).

Based on these assumptions, we formulate the following hypotheses:

— H_5_: Entrained body movement strengthens the inner representation of the temporal regularity in the music.
— H_6_: Entrained body movement increases the pleasure of listening to the music.

Musicians' entrained body movement directly affects the musical properties through the physical act of performance (thus linking the right- and leftmost boxes of Figure 1).

### How Contextual and Personal Factors Influence the Groove Experience

A series of personal and contextual aspects have an influence on the described processes whenever a person hears music. Listeners' *personal backgrounds* (Figure 1, bottom) affect their responses to music (Levinson, 1987; Thompson, 2007). For example, it may be difficult for listeners to parse the *temporal regularities* if they are unfamiliar with the music (LaBarba et al., 1992) or if the rhythm is complicated. The *interest* and the experienced *pleasure* may be strong if the music agrees with listeners' musical taste or triggers positive biographic memory (Holbrook and Schindler, 1989). A person might have a general affinity to dancing or body movement, whereas another person might be more averse to these activities (Clegg et al., 2018). Amusia and beat deafness might impede decoding and representing the regularities of the music (Dalla Bella and Peretz, 2003; Sowinski and Dalla Bella, 2013), or health issues might prevent a person from carrying out entrained body movement (Thaut and Hoemberg, 2014).

The context of the *concrete listening situation* should also be taken into consideration (Figure 1, top). For example, live performances of rock music have been found to trigger a stronger groove experience than recorded performances (Swarbrick et al., 2019). Todd and Cody (2000) suggested that music triggers a stronger reaction in the listener when it is loud as opposed to soft. In some contexts, it might be socially desirable to express an urge to move through entrained body movement, while it might be inappropriate in others (e.g., dance event vs. classical music concert). A person might be in the right mood (Hunter et al., 2011) or too tired to react to the music. We can expect a person's emotional state (Juslin and Västfjäll, 2008; Koelsch, 2014) to influence the groove experience as well. Finally, the use of psycho-active substances such as alcohol or ecstasy may lower inhibitions that would otherwise prevent a person from moving with the music (Steele and Southwick, 1985; Milroy, 1999).

Personal and contextual factors affect the groove experience in a wide variety of ways. Several of these effects have been reported in previous research, but many have not yet been thoroughly investigated. Given the diversity of these effects and their potentially complex interactions, we refrain from formulating concrete hypotheses at this stage.

## Conclusions

This opinion paper proposes a psychological model of musical groove. It postulates that musical properties affect listeners' urge to move, mediated by their inner representation of temporal regularity, by their time-related interest, and by the pleasure they experience while listening. The listening context and the listener's own musical and personal background influence this process. The urge to move may lead to entrained body movement, triggering a nuanced interplay of sensory processes and hedonic states.

Empirical work and further theoretical discussion will be required to establish whether and to what extent this paper's hypotheses hold under scrutiny. Important elements may be missing in the current model, or existing elements might prove to be irrelevant. The mental processes described in the model are purely conceptual; future work will show how they relate to processes in the neural substrate.

## Author Contributions

OS drafted the manuscript and created the figure. DR, TB, LK, FH, RJ, AB, and EA contributed to the development of the model and commented on the paper.

### Conflict of Interest Statement

The authors declare that the research was conducted in the absence of any commercial or financial relationships that could be construed as a potential conflict of interest.
